# Neonatal intensive care unit design and healthcare-associated infections: a scoping review

**DOI:** 10.1017/ash.2026.10846

**Published:** 2026-09-21

**Authors:** Pavan V. Thakkar, Natalia Josiah, Elizabeth R. Blackwood, Ganga S. Moorthy, Lakshmi Katakam, Melissa Campbell, Deverick J. Anderson, Ibukunoluwa C. Kalu

**Affiliations:** 1 Harvard University, Cambridge, MA, USA; 2 Duke Clinical Research Institute, Durham, NC, USA; 3 Advent Health, Winter Garden, FL, USA; 4 Duke University Medical Center Library and Archives, Durham, NC, USA; 5 Department of Pediatrics, Division of Pediatric Infectious Diseases, Duke University School of Medicine, Durham, NC, USA; 6 Department of Pediatrics, Division of Neonatal-Perinatal Medicine, Duke University School of Medicine, Durham, NC, USA; 7 Duke Center for Antimicrobial Stewardship and Infection Prevention, Division of Infectious Diseases, Duke University School of Medicine, Durham, NC, USA

## Abstract

**Background:**

Healthcare-associated infections (HAIs) contribute substantially to morbidity and mortality among hospitalized neonates. Many hospitals have transitioned from the traditional open-bay NICU layout to single-family room (SFR) designs. However, evidence regarding the association between unit design and neonatal HAI incidence or microbial colonization remains limited and inconclusive.

**Objective:**

We conducted a scoping review to evaluate the association between NICU design (SFR versus open-bay) and HAIs, to inform evidence-based design and infection prevention strategies.

**Methods:**

In February 2025, we searched MEDLINE via PubMed for studies on neonatal unit designs. We included English-language studies that directly compared HAI or colonization outcome measures between open and SFR room designs in neonatal units. We excluded studies that did not assess neonates, were based on outpatient settings or non-neonatal wards, used animal models, only focused on impact on families and/or healthcare workers or were summary statements or reviews.

**Results:**

Our search yielded 610 studies, of which 45 progressed to full-text review; we ultimately included 10 studies in this review all of which utilized observational designs. Eight compared outcomes before and after moving to a new ward while two compared units within the same hospital. All but three included studies were from the United States. Studies assessed a variety of infection and colonization measures, including but not limited to sepsis, catheter-associated infections, and multidrug resistant organisms, with widely varying results.

**Conclusions:**

The current evidence regarding the association between NICU design and HAIs is mixed and context dependent.

## Introduction

Healthcare-associated infections (HAIs) result in substantial morbidity and mortality among hospitalized neonates.^1^ Neonatal intensive care units (NICUs) are increasingly adopting single-family room (SFR) designs in place of open bays for optimizing care delivery and providing family centered care. These SFR designs can facilitate parent-neonate interaction,^2,3^ improve privacy,^4^ reduce noise levels,^5,6^ and increase parent and healthcare provider satisfaction.^4,5^ However, evidence regarding the impact of unit design on HAIs and colonization burden in the NICU is limited.^7^ Some studies have suggested that SFR designs may reduce the incidence of neonatal sepsis,^8^ bloodstream infections, and *Staphylococcus aureus* infections^9^ relative to open-bay NICUs. However, other studies have challenged assumptions about the inherent infection prevention benefits of SFR designs, suggesting that structural design alone does not necessarily result in reducing infections and improving outcomes. Other factors, particularly hand hygiene, are essential in infection prevention, regardless of unit design.

Minimal data exist on optimal room designs in neonatal settings. Often, pediatric built environments rely on design ideas that prioritize the health needs of adult patients. Additionally, although sustainability, temperature control, air flow, navigational needs, bed spacing, patient security, and privacy are important, the care environment’s impact on HAI incidence must be prioritized.^10^ Growing global interest in neonatal infection prevention underscores the need for robust, evidence-based guidelines regarding HAI and the neonatal environment. Therefore, we conducted a scoping literature review to evaluate the association between NICU design (single-family or open bay) and neonatal HAIs or colonization burden.

## Methods

### Information sources and search eligibility criteria

In collaboration with a medical librarian (E.B.), we conducted a MEDLINE search via PubMed for articles regarding neonatal units and unit design (SFRs and open wards) in February 2025. The full search strategy and keywords are described in Supplemental Table 1. No limits for language or publication date were applied at this stage. The study protocol was not registered. We reported results in accordance with the PRISMA extension for scoping reviews.^11^

### Selection of sources of evidence

All articles resulting from the search were imported into Covidence (Veritas Health Innovation, Melbourne, Australia) for review. Each title and abstract were screened independently by two reviewers (PVT, NJ, or ICK), with discrepancies resolved by a third reviewer (GSM or DA). Subsequently, studies selected for inclusion in the full-text review were again screened by two reviewers (PVT, NJ, ICK, or DA), with discrepancies resolved by ICK.

### Eligibility criteria

We included published English-language studies that directly compared HAI or colonization outcome measures between the different NICU designs. Specifically, we reviewed catheter or central line associated bloodstream infections (CLABSIs), catheter-associated urinary tract infections, infections and/or colonization with methicillin-resistant *Staphylococcus aureus* (MRSA), methicillin-susceptible *Staphylococcus aureus* (MSSA), or multidrug-resistant organisms (MDROs). We did not use date restrictions. We excluded studies that occurred solely in outpatient settings or non-neonatal units, only assessed patients ≥1 year of age, focused on animal models, only described the impacts of unit design on families and/or healthcare workers, or were summary statements or reviews.

## Results and discussion

### Selection of sources of evidence

**Figure 1.**
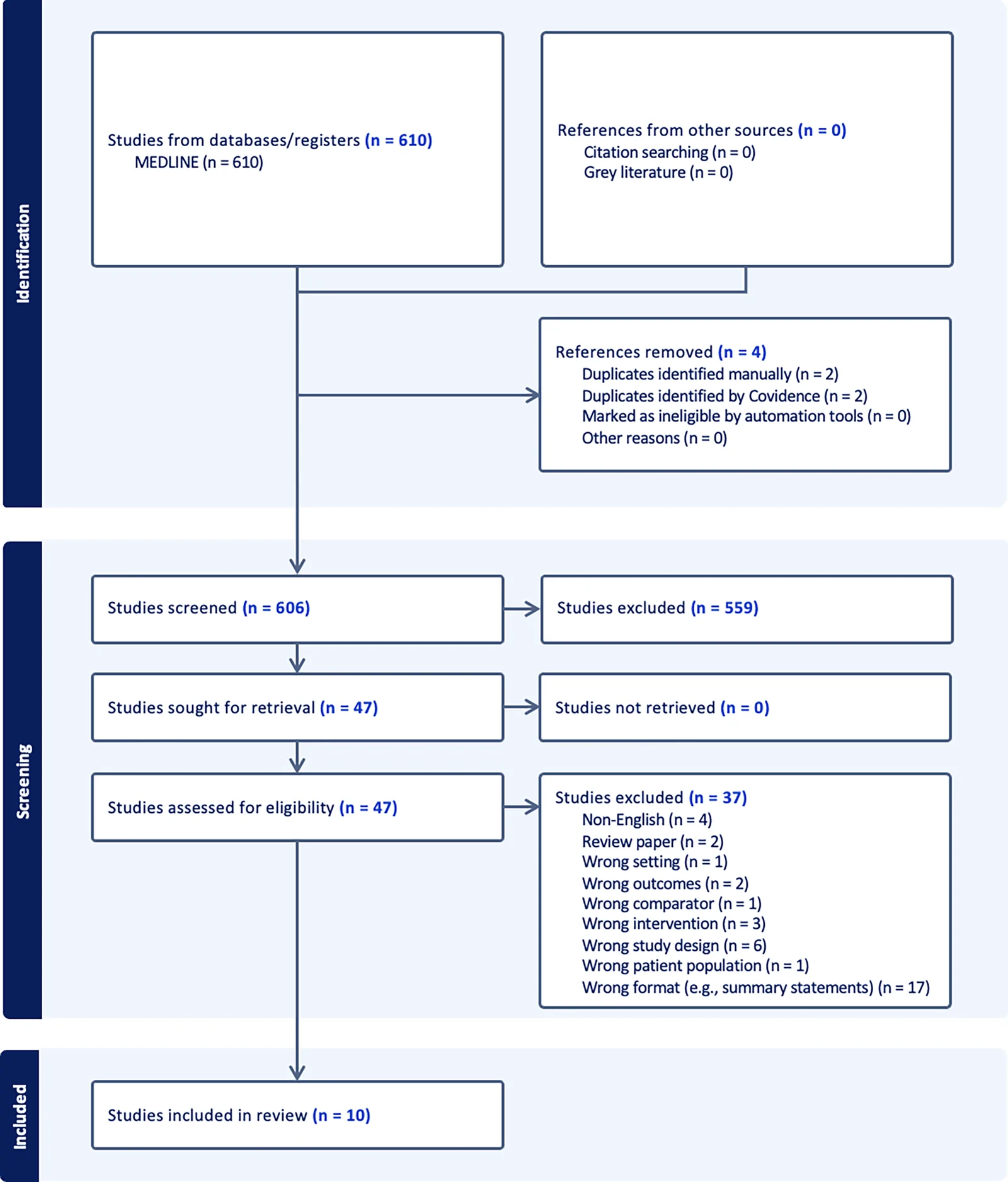
Figure 1 long description. PRISMA flow diagram.

The initial search yielded 610 studies for title and abstract review, of which four duplicates were removed. Forty-seven studies were selected for full-text review, of which 37 were excluded at this stage, leaving 10 studies included in this scoping review (Figure 1).

### Characteristics of included studies

The 10 included studies were from two countries (the United States and the Netherlands), and consisted of six retrospective cohort studies, two prospective cohort studies, one matched case-control study, and one study with unclear methodology (Table 1). Of these, eight studies compared outcomes before and after moving to a new ward, whereas two studies compared neonatal rooms with differing designs within the same hospital.

**Table 1.** Characteristics and results of 10 studies evaluating neonatal room design and Healthcare-Associated Infection (HAI) outcomes Table 1 long description.

Study	Setting	Design	N	Comparison	HAI outcomes assessed	Additional non-HAI outcomes assessed	Results: Effect of SFR on HAI outcomes*
Domanico et al. 2011^12^	Level III NICU, West Virginia, USA	Prospective cohort (before-after)	150	Open ward vs SFR NICU design	Sepsis incidence	Apneic events, mortality, sustained lactation, environmental parameters	↓ Sepsis incidence
Jansen et al. 2021^14^	Level III NICU, Netherlands	Retrospective cohort (before-after)	712	Open-bay vs SFR NICU design	HAI and CLABSI incidence, antibiotic use	N/A	= HAI = CLABSI incidence
Van der Hooven et al. 2023^19^	Level III NICU, Netherlands	Retrospective cohort (before-after)	1,195	Open-bay vs SFR NICU design	MDRO and infectious cluster incidence	N/A	= MDRO clusters
Van Veendaal et al. 2020^13^	Level II NICU, Netherlands	Retrospective cohort (before-after)	1,046	Open-bay NICU vs SFR NICU with family integrated care	Late-onset sepsis incidence	Length of stay, mediators of decreased sepsis	↓ Late-onset sepsis incidence
Walsh et al. 2006^5^	Level IIIC NICU, Tennessee, USA	Unclear (before-after)	Not Reported	Open unit vs SFR NICU design	CLABSI	Nurses’ perceptions, sound levels	↓ CLABSI
Hourigan et al. 2018^17^	Virginia, USA	Prospective cohort (before-after)	32	Shared-space vs SFR NICU design	Colonization patterns	Antibiotic resistance genes, virulence genes, environmental samples	= Viral and fungal colonization
Julian et al. 2015^15^	St. Louis, Missouri, USA	Retrospective cohort	1826	Open-unit vs single-patient NICU rooms	MRSA colonization, colonization-free survival time, late-onset sepsis, sepsis-free survival	Mortality	= MRSA colonization = Colonization-free survival times = Late-onset sepsis = Sepsis-free survival
Puumala et al. 2020^7^	Dallas, Texas, USA	Retrospective cohort (before-after)	9,995 encounters	Open-bay vs SFR NICU design	Late-onset sepsis incidence	Oral feeding, length of stay, growth	↑ Late onset sepsis incidence among term and postterm infants
Van der Hooven 2022^18^	Level III NICU, Netherlands	Retrospective cohort (before-after)	1,433 (infection), 1,293 (colonization)	Open-bay vs single-room NICU Design	Colonization with MDROs or 3G-CRB	N/A	= MDRO colonization = 3G-GRB colonization
Washam et al. 2018^9^	Cincinnati, Ohio, USA	Retrospective matched case-control	1751 total; MRSA 67 cases, 201 controls; MSSA 174 cases, 358 controls	Multibed pods vs single-bed NICU	MRSA and MSSA acquisition	N/A	↓ MRSA acquisition ↓ MSSA acquisition

*SFR*, Single Family Room; *NICU*, Neonatal Intensive Care Unit; *CLABSI*, Central Line-Associated Bloodstream Infection; *MDRO*, Multidrug-Resistant Organism; *MRSA*, Methicillin-Resistant *Staphylococcus aureus*; *MSSA*, Methicillin Sensitive *Staphylococcus aureus.*

*A significant decrease is indicated by ↓; a significant increase is indicated by ↑. The = symbol indicates no significant change in that outcome.

Four studies assessed sepsis incidence. CLABSIs, MDROs, and MRSA were each examined by two studies. Additional infection outcomes assessed by individual studies included *Klebsiella pneumoniae, Escherichia coli, Enterobacter cloacae*, MSSA, third-generation cephalosporin-resistant bacteria (3G-CRB), and nonspecific HAIs.

### Sepsis

An important infectious outcome and high-risk factor for neonatal morbidity is sepsis. Yet, studies reported conflicting findings regarding the association between NICU design and sepsis incidence. One prospective single center study estimated that unadjusted neonatal sepsis incidence was reduced from 11% (14 cases) in the open-bay unit (OBU) to 6% (6 cases) in the SFR unit.^12^ Similarly, transitioning from OBU to SFR was associated with decreased odds of late-onset sepsis (OR 0.486, 95% CI 0.293–0.807), and decreased length-of-stay in a Dutch level II NICU.^13^ Notably, this study identified decreased parenteral nutrition following implementation of family-integrated care in SFRs as a primary mediator of this association.^13^ Conversely, a 5-year study of nearly 10,000 NICU encounters in a public safety-net hospital found no significant change in sepsis incidence before and after a switch from OBU to SFR.^7^ Furthermore, in the same study, although a decline in sepsis incidence was observed among extremely preterm infants following the transition to a SFR design, the change was associated with an *increase* in sepsis incidence among term and postterm infants.^7^ In sum, two studies found SFRs decrease sepsis incidence, whereas one study found no significant change, and another study found increased sepsis among term and postterm infants in SFRs. These contrasting findings may be due to changes in staffing patterns, other parallel quality improvement efforts targeting prematurity, feeding regimens, or data availability for sepsis diagnoses. Nevertheless, these findings suggest that architectural factors alone may not broadly improve patient outcomes, but there may be subsets of patients for whom NICU design elements may offer an advantage.

### CLABSIs

Findings on the association between NICU design and CLABSIs were similarly mixed. One study reported a CLABSI decrease from 10.1 per 1,000 device days in the OBU to 3 per 1,000 device days in SFRs.^5^ However, this study was primarily focused on nurses’ perceptions of the ward design and lacked methodological information regarding the neonatal population used to calculate incidence. In contrast, a rigorous Dutch study that performed univariate and multivariate analyses of more than 700 infants born at <32 weeks gestational age found no statistically significant difference in HAI incidence, including CLABSI, upper respiratory tract infections, urinary tract infections, or other outcomes such as necrotizing enterocolitis and antibiotic use between the OBU and SFR groups.^14^

### MRSA and MSSA colonization

To broaden our review, we included studies that evaluated colonization in the NICU. Surprisingly, we found that SFR design may increase colonization rates in neonates. Although one single-center retrospective study in a hospital with SFRs and OBUs found no significant difference in MRSA colonization rates, late-onset sepsis, or mortality between infants cared for in the two layouts, they noted that higher average daily census was associated with increased MRSA colonization only in the SFR (hazard ratio 1.31, 95% CI 1.02–1.68).^15^ Across both unit configurations, the only factor that consistently reduced MRSA colonization was hand hygiene compliance upon entry and exit.^15^ This finding is consistent with a 2023 quality improvement study, identified while reviewing supporting literature, that found an *increase* in MRSA infection and colonization after transitioning to a SFR design, which was hypothesized to be associated with reduced hand hygiene in the SFR NICU.^16^ Additionally, one of the included single-center studies that performed metagenomic sequencing on stool and groin swabs from infants before and after transitioning from an OBU to SFR NICU found that infants in the SFR harbored a higher abundance of antibiotic resistance and virulence genes despite lower overall antibiotic usage.^17^ Although the individual bacterial, viral, and fungal taxa were similar between cohorts, skin microbial profiles differed significantly between groups (*P* = .0001).^17^

### MDROs

In another challenge to the assumption that SFRs may be protective for infants, a single-center study that collected weekly throat and rectal surveillance cultures from 1,293 infants during the two years before and after transitioning from an OBU to SFR design found that the transition to SFRs did not significantly reduce rates of colonization with MDROs or 3G-CRB.^18^ Moreover, in a 2-year period, another single-center study in the Netherlands identified one MDRO cluster among four patients in an OBU and four MDRO clusters among nine patients following the change to a SFR design.^19^ Together, these findings challenge the assumption that SFRs may be protective for infants and highlight potential multifactorial reasons for these patterns. Reasons include staffing model changes related to SFRs, Hawthorne effect in open bay settings which may improve hand hygiene compliance, limited direct exposure to the environment in open bay settings due to space restrictions, and population-level changes to MDRO prevalence.

### Summary of findings

Ultimately, in this scoping review of 10 observational studies, we found mixed and conflicting evidence regarding the association between NICU design and HAIs. Despite limited comparative analyses in the included studies, there were general reports of a decline in incidence of infectious outcomes when NICUs were converted from open bay to SFR, however the data interpretation relied heavily on the context and HAI outcome of interest. These contrasting findings highlight the need for rigorous research to test assumptions about the inherent infection prevention benefits of SFRs and underscore the importance of rigorous hand hygiene practices, staffing, and environmental cleaning, irrespective of unit design. What these studies clearly establish is that structural design alone does not guarantee better HAI outcomes.^15,18^

### Limitations

Our study has several limitations. First, we excluded non-English publications which may have limited our search to healthcare settings with more resources and fewer OBUs. However, the paucity of data on this topic and our detailed search strategy suggests that we may have captured most of the available literature focused on NICUs. Secondly, our review incorporated multiple infectious outcomes including sepsis, colonization, and HAIs typically included in infection prevention surveillance. Limiting outcomes to only HAIs or CLABSIs or device-associated infections may have shown more consistent associations with NICU design. Additionally, all included studies were observational, making it difficult to account for confounders that mitigate the relationship between unit design and HAIs. Many of these studies were also before and after comparisons, in which practice or workflow changes during the unit transition may have played a role in HAI incidence. For example, physical expansions in NICU space may be associated with a corresponding increase in patient volume; therefore, outcomes attributed to the design change may reflect staffing adjustments or differences in patient population driven by increased capacity. Additionally, as the threshold for active resuscitation has shifted toward lower gestational age, it is possible that the “after” cohorts may have been systematically different and of higher acuity than the “before” cohorts, due to the evolution of neonatology practice, rather than physical changes in unit structure.

To ameliorate these limitations, future studies should consider controlling for gestational age distribution and illness severity across study periods, utilizing risk-adjusted outcome metrics, tracking unit census data as a covariate, and using concurrent controls when possible.

## Conclusion

Overall, the evidence linking SFR design to infectious and colonization outcomes remains mixed and context-dependent for NICUs. Additional studies delineating the impact of unit design on HAIs and other neonatal outcomes are needed. Further research, such as longitudinal and multi-center studies may help to clearly delineate the relationship between NICU design, HAIs, microbial colonization and other neonatal outcomes.

## Supporting information

10.1017/ash.2026.10846.sm001 Thakkar et al. supplementary material Thakkar et al. supplementary material

## Supplementary material

The supplementary material for this article can be found at https://doi.org/10.1017/ash.2026.10846.

## Figure 1. Long description

The flowchart begins with the identification phase, where 610 studies are sourced from databases/ registers, specifically MEDLINE. No references are obtained from other sources such as citation searching or grey literature. Four references are removed due to duplicates identified manually and by Covidence. In the screening phase, 606 studies are screened, with 559 studies excluded. Forty-seven studies are sought for retrieval, and all are successfully retrieved. These 47 studies are assessed for eligibility, with 37 studies excluded for various reasons including non-English language, review paper status, wrong setting, wrong outcomes, wrong comparator, wrong intervention, wrong study design, wrong patient population, and wrong format. Finally, 10 studies are included in the review.

Navigate back to Figure 1..

## Table 1. Long description

A table with 10 rows and 7 columns comparing studies on neonatal room design and healthcare-associated infection outcomes. Columns include Study, Setting, Design, N, Comparison, HAI outcomes assessed, Additional non-HAI outcomes assessed, and Results: Effect of SFR on HAI outcomes. Each row provides details for a specific study, including the study name, location, study design, number of participants, comparison groups, healthcare-associated infection outcomes assessed, additional non-HAI outcomes assessed, and the results of the study on HAI outcomes. The table captures various studies conducted in the United States and the Netherlands, with different study designs and comparisons.

Navigate back to Table 1..
