# Supplementary material for: Neonatal intensive care unit design and healthcare-associated infections: a scoping review
Source: Antimicrob Steward Healthc Epidemiol. 2026 Sep 21;6(1):e285. doi: 10.1017/ash.2026.10846 (PMC13760403; doi:10.1017/ash.2026.10846)
Supplement: Supplementary file 1 [file S2732494X26108468sup001.docx]

**SUPPLEMENTAL MATERIAL**

**Supplemental Table 1. Search Strategies**

**MEDLINE (via PubMed)
Search date: 2/11/2025**

| Concept | Strategy | Results |
| --- | --- | --- |
| #1 Neonatal Wards | "Intensive Care Units, Neonatal"[Mesh] OR "Nurseries, Hospital"[Mesh] OR "Neonatal intensive care"[tiab] OR NICU[tiab] OR "neonatal ward"[tiab] OR "neonatal wards"[tiab] | 43,394 |
| #2 Single – Family Rooms | "Rooming-in Care"[Mesh] OR "single family room"[tiab] OR "single-family room"[tiab] OR "single family rooms"[tiab] OR "single-family-rooms"[tiab] OR "single family rooms"[tiab] OR "single-family-rooms"[tiab] OR "hospital room"[tiab] OR "hospital rooms"[tiab] OR "single room"[tiab] OR "single rooms"[tiab] OR "single bed"[tiab] OR "single beds"[tiab] OR "single-bed"[tiab] OR "single-beds"[tiab] OR "private room"[tiab] OR "private rooms"[tiab] | 2,810 |
| #3 Open Ward | "open-bay"[tiab] OR "open-bays"[tiab] OR "open bay"[tiab] OR "open bays"[tiab] OR "ward setting"[tiab] OR "shared room"[tiab] OR "shared rooms"[tiab] OR multibed[tiab] OR "multi-bed"[tiab] OR "multiple bed"[tiab] OR "multiple beds"[tiab] OR "multiple-bed"[tiab] OR "multiple-beds"[tiab] OR "dual occupancy"[tiab] OR "dual-occupancy"[tiab] OR "open unit"[tiab] OR "open-unit"[tiab] OR "pod"[tiab] OR "pods"[tiab] OR "open-pod"[tiab] OR "open pod"[tiab] OR "open pods"[tiab] OR "open-pods"[tiab] | 21,725 |
| #4 Ward Design | "Facility Design and Construction"[Mesh] OR "Health Facility Environment"[Mesh] OR "ward design"[tiab] OR "ICU design"[tiab] OR "NICU design"[tiab] OR "intensive care unit design"[tiab] OR "room design"[tiab] OR "interior design"[tiab] | 32,569 |
| #5 Comparison | #2 OR #3 OR #4 | 56,336 |
| #6 Without Maternity Terms | #1 AND #5 | 610 |
| #7 Validation String | #6 AND (31911646 OR 29157314 OR 29355578 OR 28780548) | 3/4 |
